# “I felt special!”: a qualitative study of peer‐delivered HIV self‐tests, STI self‐sampling kits and PrEP for transgender women in Uganda

**DOI:** 10.1002/jia2.26201

**Published:** 2023-12-26

**Authors:** Andrew Mujugira, Beyonce Karungi, Jackson Mugisha, Agnes Nakyanzi, Monica Bagaya, Brenda Kamusiime, Alisaati Nalumansi, Grace Kakoola Nalukwago, Vicent Kasiita, Chris Collins Twesigye, Olivia Nampewo, Rogers Nsubuga, Kikulwe Robert Nyanzi, Timothy Muwonge, Monique A. Wyatt, Norma C. Ware, Jessica E. Haberer

**Affiliations:** ^1^ The Infectious Diseases Institute Limited Makerere University Kampala Uganda; ^2^ Department of Global Health University of Washington Seattle Washington USA; ^3^ Transgender Equality Uganda Kampala Uganda; ^4^ Department of Global Health and Social Medicine Harvard Medical School Boston Massachusetts USA; ^5^ Harvard Global Cambridge Massachusetts USA; ^6^ Department of Medicine Brigham and Women's Hospital Boston Massachusetts USA; ^7^ Center for Global Health Massachusetts General Hospital Boston Massachusetts USA; ^8^ Harvard Medical School Boston Massachusetts USA

**Keywords:** peer delivery, HIV self‐testing, sexually transmitted infections, self‐sampling, pre‐exposure prophylaxis, Africa

## Abstract

**Introduction:**

Peer delivery is a client‐centred approach that could maximize the coverage and impact of HIV services for transgender women (TGW). We conducted qualitative interviews to examine how peer‐delivered HIV self‐testing (HIVST), sexually transmitted infection self‐sampling (STISS) and oral pre‐exposure prophylaxis (PrEP) influenced prevention choices among TGW and their intimate partners in Uganda.

**Methods:**

Within a cluster randomized trial of peer‐delivered HIVST, STISS and PrEP among HIV‐negative TGW (NCT04328025), we conducted 55 qualitative interviews with 30 TGW, 15 intimate partners and 10 TGW peers (August 2021–February 2022). TGW interviews explored: (1) HIV self‐test and PrEP experiences; (2) HIVST with intimate partners; and (3) descriptions of self‐sampling for STI testing. Partner interviews covered: (1) experiences with HIVST; (2) disclosure of HIV status to intimate partner; and (3) descriptions of sexual behaviours after testing. Peer interview topics included: (1) intervention delivery experiences; and (2) recommendations for peer‐delivered HIV prevention services to TGW, including psychological support and coping strategies. Qualitative data were analysed using an inductive content analytic approach.

**Results:**

Peer‐delivered combination prevention was valued by this group of TGW and their partners. (1) Peer services extended beyond delivering HIV/STI kits and PrEP refills to caring for individual health and wellbeing by providing stigma coping strategies. Peer psychosocial support empowered research participants to become “HIVST ambassadors,” teach non‐study TGW about self‐testing and PrEP, and encourage linkage to care. (2) HIVST with intimate partners and mutual disclosure of HIV status strengthened partnered relationships. PrEP use after both partners tested HIV negative implied infidelity. (3) Self‐sampling enabled TGW to take control of their STI testing and avoid the embarrassment of exposing their bodies. Privacy and confidentiality motivated the uptake of STI testing and treatment.

**Conclusions:**

In this sample of TGW from Uganda, peer delivery of HIVST, STISS and PrEP refills benefitted individual prevention efforts and extended to a new linkage of TGW not engaged in care. Integrating peer services into differentiated PrEP delivery could increase HIV/STI test coverage and PrEP use in this vulnerable population.

## 1 INTRODUCTION

Globally, transgender women (TGW; people who are assigned male sex at birth but identify as female) are 14 times as likely to be living with HIV as other women [1]. The pooled odds for HIV in sub‐Saharan Africa are 1.6 times greater among TGW than men who have sex with other men [2]. The HIV vulnerability of TGW is driven by high rates of receptive condomless anal sex, multiple sexual partnerships, sex work with cisgender men, intimate partner violence and criminalization [3, 4, 5, 6, 7]. However, TGW are less likely than the general population to seek HIV services primarily because of multiple intersecting individual, interpersonal and structural stigmas related to race, gender and sexual orientation, and discrimination in healthcare settings [8, 9, 10]. HIV‐related intersectional stigma and discrimination is a critical and complex barrier to the uptake of evidence‐based HIV prevention and treatment interventions [11]. The Joint United Nations Programme on HIV/AIDS (UNAIDS) target is for 95% of people at risk of HIV infection, such as TGW, to use “appropriate, prioritized, person‐centred and effective combination prevention options by 2025” [12]. Still, this goal is unlikely to be reached with current approaches.

Differentiated service delivery (DSD) is a client‐centred, contextually appropriate approach that aims to tailor HIV services to the needs and preferences of clients while optimizing clinical outcomes and reducing health system burden [13]. Peer delivery could maximize the coverage, effectiveness, efficiency and impact of HIV services for TGW [14, 15] and is recommended by the World Health Organization for HIV testing and delivery of pre‐exposure prophylaxis (PrEP) refills after clinic‐based initiation [16, 17], particularly for hidden populations. HIV self‐testing (HIVST) may reach TGW who may otherwise not test or test as frequently by providing autonomy, freedom and control over testing decisions [18, 19, 20]. Using HIVST to support oral PrEP delivery was acceptable, feasible and preferred to facility‐based testing for TGW in Uganda [21]. Self‐sampling for sexually transmitted infection (STI) testing significantly increases testing coverage and detection of bacterial STIs [22]. It is feasible, acceptable and empowering in resource‐limited settings [23] for TGW with high STI burden [24]. Thus, peer delivery of HIVST, STI self‐sampling (STISS) and PrEP could decentralize prevention services and reach TGW not engaged in care.

To our knowledge, no published qualitative studies have evaluated how peer delivery of HIVST, STISS and PrEP could motivate uptake by TGW and their intimate partners. Within a randomized trial designed to assess the impact of HIVST and STISS as facilitators of peer‐delivered PrEP, we conducted a qualitative study to examine prevention choices of TGW in Uganda.

## 2 METHODS

### 2.1 Population and procedures

The Peer Study was a pilot cluster randomized controlled trial (RCT) that evaluated the impact of HIVST and STISS on peer‐delivered PrEP to TGW in Kampala, Uganda, between October 2020 and July 2022 (ClinicalTrials.gov: NCT04328025). Using Research Electronic Data Capture (REDCap) software [25], 10 TGW peer groups (each comprised of one peer and eight TGW) were randomized 1:1 to either monthly peer delivery of HIVST, STISS and PrEP (intervention) or quarterly in‐clinic HIV/STI testing and PrEP prescription (standard of care [SOC]). This project employed a community‐based participatory research approach [26] that involved partnering with TGW civil society organizations, consulting TGW during the grant and protocol writing process, identifying and training TGW peers to lead recruitment and retention activities, as well as including TGW on both the research team and community advisory group. Potential power dynamics were discussed to ensure the active involvement of TGW throughout the trial.

All TGW were enrolled at the research clinic where they started PrEP, attended quarterly clinic visits and were followed for 12 months. At each study visit, they received HIV and STI testing, free treatment for *Neisseria gonorrhoeae* (NG) and *Chlamydia trachomatis* (CT), individualized HIV risk reduction counselling, free condoms and lubricant as SOC. They also received four OraQuick® HIV self‐test kits (OraSure Technologies, USA) for themselves or use with intimate partners, that is persons in committed relationships with TGW. Three bottles of PrEP medication (lamivudine/tenofovir disoproxil fumarate; 3TC/FTC), each with a 1‐month supply, were dispensed at quarterly visits to all participants. PrEP adherence was measured using tenofovir diphosphate levels in dried blood spot samples, assessed quarterly and batch tested [27].

TGW peers identified during formative research were trained in intervention delivery by study staff for 2 weeks for the peer delivery intervention. The training curriculum was derived from formative research results and included HIV self‐test use and interpretation of results, self‐collection of oro‐pharyngeal, rectal and urine specimens using visual guides for self‐collected swabs, HIVST and STISS kit delivery, PrEP refills and linkage of TGW who tested positive into HIV care. Participants were introduced to TGW peers at enrolment and phone numbers were mutually shared with permission. Peers were given study phones for calling participants, and pre‐paid voice/data bundles were loaded monthly. Each month between quarterly visits, trained peers phoned TGW in their group to schedule individual visits at a place of their convenience (e.g. home, shelter, workplace) in the months between quarterly visits. The peer intervention consisted of: (1) delivery of additional HIV self‐test kits and PrEP refills; (2) teaching TGW how to use STI self‐sampling kits to self‐collect oro‐pharyngeal, rectal and urine specimens, with same‐day specimen delivery to the research clinic; and (3) psychosocial support for PrEP adherence, HIVST with intimate partners and peer counselling (emotional and social support, self‐efficacy and social inclusion) following national guidelines [28]. All participants received free STI testing and treatment for CT and NG. HIVST, STISS, PrEP adherence and sexual risk behaviours were assessed quarterly using semi‐structured questionnaires. All participants received individualized HIV risk reduction counselling, free condoms and lubricant as SOC. Intervention design was guided by the Social Ecological Framework for PrEP introduction [29].

### 2.2 Sampling and recruitment

During follow‐up visits for the cluster RCT, we invited a random sample of 30 TGW in the peer delivery arm, 15 of their intimate partners and all 10 peers involved in intervention delivery to participate in a qualitative study. Study staff contacted TGW participants and peers to schedule interviews. Intimate partners were invited to participate in interviews through their TGW partners in the research study, who provided their contact details.

### 2.3 Data collection

Qualitative data collection began in August 2021 and ended in February 2022. It consisted of a single, in‐person interview with each of the 30 participating TGW, 15 intimate partners and 10 peers (Total: 55 interviews). Interviews with TGW covered: (1) experiences of HIVST; (2) how HIV self‐tests were used with intimate partners; (3) descriptions of sample self‐collection for STI testing; and (4) views on peer delivery of test kits and PrEP refills. Partner interviews explored: (1) experiences with HIVST; (2) disclosure of HIV status to intimate partner(s); (3) descriptions of sexual behaviours after testing; and (4) use of antiretroviral treatment (ART) or PrEP after testing. Peer interview topics included: (1) experiences of delivering test kits and PrEP refills; (2) perspectives of collecting samples for STI testing; and (3) recommendations for peer‐delivered HIV prevention services to TGW. Experienced social scientists (cisgender women and men) conducted all interviews in English or Luganda (local language) at locations of the participant's choice where conversations could not be overheard (interview guides are included in the Supplementary Appendix). Interviews were about 60 minutes long, audio‐recorded with the participant's permission and transcribed in English by the interviewer from recordings. We performed quality checks for each transcript, with corrections and revisions to errors identified. Each participant received an Institutional Review Board‐approved reimbursement of UGX 30,000 (US$8.10).

### 2.4 Data analysis

We used an inductive, content analytic approach to data analysis [30]. Analysis began with repeated reviews of interview transcripts for content on experiences of peer delivery of HIVST, STISS and PrEP. Open coding, in which relevant content is delineated and provisionally labelled, was iteratively carried out by six coders (AM, AN, BK, CCT, JM and VK) to identify specific text sections. Provisional labels were defined, illustrated to become codes and assembled into a codebook. We used the codebook to code the data, with Dedoose software (SCRC, Hermosa Beach, CA) organizing the coding process. At the end of the coding process, we used codes to sort the data to suggest concepts corresponding to HIVST, STISS and PrEP use. Content categories were developed from the initial concepts. Each category includes a descriptive label, elaborative text and interview quotes illustrating the concept. Categories were derived inductively and represent all the primary themes identifiable in the data; we achieved saturation. The COREQ checklist was used for reporting study findings [31].

### 2.5 Ethics approval

The study was approved by the Mildmay Uganda Research Ethics Committee (0304‐2019), Partners Human Research Committee (Massachusetts General Hospital; 2019P001620) and Uganda National Council for Science and Technology (HS390ES). Each qualitative study participant provided separate written informed consent in English or Luganda.

## 3 RESULTS

### 3.1 Participant characteristics

At enrolment, the median age was 21 years for the 30 TGW participants (interquartile range [IQR] 20–24), 25 years (IQR 22–26) for the 15 intimate partners and 24 years (IQR 23–27) for the 10 peers (Table 1). The median schooling for TGW was 11 years (IQR 9–13). They reported a median monthly income of UGX 200,000 ($53.99); at the time of the study, Uganda's average monthly income was UGX 416,000 ($107.74) [32]. TGW reported a median of 10 anal sex acts (IQR 5–15) in the prior month. Most (27; 90%) had access to lubricants, 25 (83%) reported having access to condoms and 20 (70%) engaged in sex work. The median charge for anal sex was UGX 50,000 ($13.50) with a condom and UGX 62,500 ($16.90) without a condom, respectively. Twenty‐eight TGW (93%) had ever taken alcohol.

**Table 1:** Baseline characteristics of TGW participants (*N* = 30)

Characteristic	Median (IQR) or *N* (%)
Age (years)	
<25	25 (83)
≥25	5 (17)
Education (years)	
≤10	11 (37)
>10	19 (63)
Monthly income (UGX)	200,000 (100,000, 300,000)
Partnership status	
Intimate partner	20 (67)
No intimate partner	10 (33)
Anal sex acts, prior month (*n* = 21)	10 (5,15)
Access to condoms	25 (83)
Access to lubricants	27 (90)
Sells sex for money or goods	21 (70)
Charge for anal sex with a condom (*n* = 11)	UGX 50,000 (30,000, 100,000)
Charge for anal sex without a condom (*n* = 10)	UGX 62,500 (40,000, 200,000)
Currently taking alcohol	20 (67%)
Currently taking tobacco	2 (7%)
Currently using recreational drugs	1 (3%)
Ever used hormone replacement therapy (*n* = 16)	0 (0%)
Ever incarcerated	7 (23%)
Ever incarcerated for being transgender (*n* = 7)	3 (43%)

*Note*: Quantitative data derived from parent clinical trial.

Abbreviations: IQR, interquartile range; UGX, Uganda Shillings.

### 3.2 Qualitative results

We present three categories that illustrate how TGW participants, their intimate partners and study peers experienced peer‐delivered combination HIV prevention services and how care was tailored to each individual's needs in the context of differentiated PrEP delivery. The first category shows the positive impact of peer support for HIV/STI testing by TGW. The second shows how intimate partners experienced HIVST. The third describes how the novelty of self‐collecting STI samples empowered TGW and facilitated STI testing and treatment. Overall, TGW experienced peer delivery of HIVST, STISS and PrEP as improving their prevention uptake, strengthening their intimate relationships and changing their sexual behaviours while reducing stigma.

#### 3.2.1 Category 1: Peer delivery experiences

##### 3.2.1.1 Peer services were welcomed and valued as a trusted way to receive care and build community

TGW welcomed the novelty of peer services, which were unavailable to the general population. They valued peer support because it extended beyond delivering HIV prevention commodities to caring for their health and wellbeing. Peer counselling motivated TGW to take PrEP to demonstrate good adherence at the next peer visit. Self‐collection of samples was valued in a setting where STI testing was not routine outside research studies, and self‐sampling was unavailable to the public.

*“I felt special! Somebody has come to me to ask me how I am? It is a new thing! It is unique. I pray that it continues. It is a reminder that my peer is coming, so I must take my pills, and when he comes back, I have to show him an empty [bottle]. He asks you about PrEP. ‘Have you been taking PrEP’? That care he gives you when he is asking. And nobody is going to do that for you. Someone has brought you kits which are expensive. The general population can't get them, and am getting this.” (TGW, age 19)*

Some TGW were initially hesitant about trusting the peer delivery system, particularly the ability of peers to keep personal health information confidential. The success of the peer delivery model depended on building trust between peer and client. Once peers were found trustworthy, participants gradually felt comfortable dealing with them and receiving the services they provided. As explained by one TGW: “With time, we got to understand each other, build the trust.” The peer delivery experience was viewed positively by TGW because it provided psychosocial support and facilitated access to HIV/STI care.

*“The first time the peer came to my home, I didn't know him, but he reached me on phone. Since it was my first time to meet him, I was a bit afraid. ‘Can he keep someone's information confidential?’ So, when it is his first time to come to you, you cannot open up everything to him. But the more he continues to come, you realize he is the right person. The peers are good people. They are confidential. Ever since they started working, you cannot hear anyone accusing the peer of having disclosed her information. Even checking on us all the time is a blessing.” (TGW, age 22)*

Peers observed that study participants reached out to TGW not involved in the study or engaged in care and taught them how to self‐test. Those interested in taking PrEP were introduced to the peers. This outreach had broader unanticipated benefits to the community, for example a sense of solidarity and oneness, that extended beyond the provision of HIV prevention commodities and psychosocial support to individuals under their care.

*“Study participants have helped discover other transgender women who live in the closet. Our clients have played a crucial role in sensitizing other people in our community about self‐testing. They have been our ambassadors regarding the self‐testing kit. We have been giving them two self‐tests whenever we visit them. They followed the instructions on how to self‐test the way we taught them. They have been testing their colleagues. When they come across a trans who wants to initiate PrEP, they link that person to us. That means we have played our role as peers.” (Peer, age 27)*

##### 3.2.1.2 Some peer visits created awkward situations and risked inadvertent disclosure of gender identity

TGW who lived with their parents or moved in with them during the COVID‐19 pandemic found it hard to explain the presence of a stranger and the purpose of their visit to family members in a setting where health workers did not routinely conduct home visits. Peers concurred that visiting study participants at their parents’ homes could be awkward. TGW opted to offer partial information, conceal the true purpose of the peer visit and choose another venue for peer visits (i.e. a partner's house) to avoid inadvertent disclosure of gender identity to family members. However, this strategy created new problems when intimate partners thought that peers were potential rivals.

*“The first time he came to my parents’ home, I had to create a story [for] my parents to get convinced. I had to sneak him in my bedroom, so everything was very hectic. They started asking me the kind of healthcare worker who enters my bedroom. So, I told my mum that he didn't have any problem. He just wanted some samples from me. But right now, I no longer take him to our home. I meet him at my boyfriend's place. If he wants to get samples from me, he finds me there. Though it is a bit tricky because he might think that he is my man and I have brought him to his house. The first time I told him that a peer is going to come and meet [me] here at your place, he was frustrated, but right now, he is okay with it because they know each other.” (TGW, age 24)*

*“It becomes hard for you as a peer to go in someone's home and ask for permission to work on her child. She will ask you several questions like, ‘Who are you?’” (Peer 04, age 24)*

Some peer visits were arranged at places of work. Peer delivery services were inconvenient when the peer visited the client at their workplace or called to request a visit when the client could not receive them because co‐workers were present. Picking up samples for STI testing was challenging if privacy was limited at the workplace, or the peer was unavailable when the participant could provide samples.

*“At times it is a good strategy, but we encounter difficulties with it. Sometimes the peer may come when you are at work. You look for the way of giving him the samples but in vain. You are seated in between other people. So, you really find [it] a challenge. Sometimes he can call you and you say, ‘[I] am not around.’ You are around, but you have nothing to do. Sometimes, when you are free, you can call him and say, ‘You come, there are no people…but he is already attending to others.’ So, you find it hard getting a convenient time to meet.” (TGW, 22 years)*

##### 3.2.1.3 Peer services helped avoid stigma in healthcare settings and provided support for adherence

TGW appreciated peer services because they helped them circumvent the stigma experienced at health facilities and avoid intrusive questions such as, “Why are you like this [transgender]?” Receiving HIV services in the comfort of their homes or at another convenient venue helped avoid uncomfortable experiences.

*“Peers work on us very well when they meet us in the field. It prevents us from being stigmatized in the community. Sometimes you can go to the clinic, and people start finger‐pointing. They judge you. So, if the peer meets you at your home, he would have saved you from all those illicit talks…they are so helpful to us.” (TGW, age 22)*

Peer support was valued because “if am a trans and a fellow transwoman is giving me these services, there is so much trust because I know the person who works on me understands what [I] am going through.” Peers provided psychosocial support during monthly visits and acknowledged that “adherence to PrEP is very hard.” Interacting with a peer who was also taking antiretroviral drugs enabled TGW to deal with side effects and stigma.

*“I felt bad. I thought I was HIV‐positive because there is no difference between you and the person who is positive. We are all on tablets. I was so disturbed. It [PrEP] traumatized me. I take it in the morning, and I lose appetite. I don't want to eat, sometimes it gives me headache. ‘Why am I doing this?’ I told my peer, ‘You know what, am getting out of this study.’ He said, ‘You know what, you shouldn't do that. It is not good because I personally am HIV‐positive; I don't want you to get HIV/AIDS at that age of yours.’ So, it gave me confidence and encouragement.” (TGW, age 24)*

#### 3.2.2 Category 2: HIV‐self‐testing with intimate partners

##### 3.2.2.1 Mutual partner testing strengthened relationship trust

TGW participants were encouraged to distribute HIV self‐tests to their intimate partners and use their discretion to decide when (and with whom) to test. Intimate partners unsure of their HIV status were hesitant and apprehensive when approached to self‐test for HIV. TGW countered that testing with mutual status disclosure was good for the partnered relationship. The discovery of HIV‐negative status by both partners strengthened their relationship.

*“I was afraid because it is not easy for someone to come to you and say that I want us to self‐test. I was not sure of my HIV status. She [said] I love you so much, but let's first self‐test and know our status. I asked her, ‘Why have you decided to do this?’ She said ‘I am doing it for the betterment of our lives.’ She gave it to me, and each one used their own kit. My heartbeat was fast. ‘What if results come out when am HIV‐positive? What am I going to do?’ But with God's mercy, results showed that I was HIV‐negative. Even hers was HIV‐negative. We now trust one another.” (TGW partner, age 20)*

TGW partners were relieved after knowing their HIV status: “If anyone tests HIV‐negative, it is the most precious thing.” However, PrEP use by study partners after mutual status disclosure aroused suspicion of infidelity.

*“When I tested myself and I was HIV‐negative, I didn't bother to take PrEP. But my partner is the one who takes it. I would always ask her, ‘Why do you take PrEP when you know that we are both HIV‐negative’? She would give some excuses. But I would ask myself, ‘Why is she taking PrEP’? Then I asked her, ‘Could you be having other partners?’” (TGW partner, age 25)*

##### 3.2.2.2 HIV testing motivated change in sexual behaviour

Knowledge of HIV status after self‐testing appeared to influence sexual behaviours by reducing the number of intimate partners and/or sex without a condom. Those with multiple partners stayed faithful to their study partner “because she has been there for me.” Knowledge of HIVST influenced sexual decision‐making with potential partners: “If there is no self‐test kit, let's use a condom.”

*“I no longer sleep with everybody. I closed that chapter. If I happen to sleep with someone and it is by emergency, you don't have a condom, I cannot do it. I prefer sleeping with someone when I know her HIV status.” (TGW partner, age 19)*

#### 3.2.3 Category 3: Self‐sampling for STI testing was empowering

##### 3.2.3.1 Self‐collection of samples for STI testing was challenging but ultimately rewarding

Participants who had not previously been tested for STIs were eager to collect their samples and receive their results. This was a new experience in a setting where STI testing is not routine. Obtaining test results from study staff over the phone added to the novelty of the self‐sampling experience.

*“It is me who collected my own samples. Then I brought them to the doctor. I was so eager to know my status because I had never tested before. So, when results came out, musawo [nurse] called me and told me everything. Then I was happy.” (TGW, age 20)*

Rectal sample collection was painful and uncomfortable, leading to a desire not to repeat this process. However, participants engaged in sex work decided to endure the discomfort of rectal self‐swabbing because they desired to know their test results and receive treatment if it was indicated.

*“The one I had to collect from the anus hurt. I felt pain, so I ended up spending an hour in the bathroom. I thought that I would not use it again, but when the peer brought it home, I rethought using it because I knew that I was still in the sex work business, so I had to take care of my life.” (TGW, age 25)*

##### 3.2.3.2 STI testing needed to be private and confidential

Collection of samples for STI testing by research staff during quarterly clinic visits for all intervention and SOC participants elicited feelings of shame and embarrassment: “I feel shy getting naked in front of a health worker.” Self‐collection of samples at home provided the privacy desired by TGW but involved a third party (the peer) taking them to the clinic laboratory which could breach confidentiality. TGW preferred that peers pick up and deliver samples while maintaining confidentiality.

*“I don't prefer coming to the clinic. Sometimes, I feel ashamed [about] how they take samples. But if they take my sample, at least I know the results. At first, I used to get shy but now through my education with my peer, the relationship that I have with my peer, it has since helped me. I now know that if I give him my samples, he doesn't say anything about it and he is always free with me.” (TGW, age 24)*

Self‐collection was perceived as empowering because it made TGW feel they were in control and did not have to undress for a clinician to collect rectal swab samples.

*“I will not come here in front of a health worker to get naked so that we can get the sample. I prefer doing it myself. It is good because it gives me self‐confidence. I feel that I have control over my life and privacy.” (TGW, age 23)*

##### 3.2.3.3 Preferences for receipt of STI results and treatment from a health worker

TGW preferred to receive STI test results directly from the health worker without peer involvement to maintain confidentiality. They desired that peers deliver test kits but not communicate results or deliver STI medication for confidentiality reasons.

*“When I was tested for STI, it is not my peer who informed me. He didn't even get to know about it. It was between me and musawo [nurse]. It is musawo herself who came with my file. She told me that we tested you and you were positive…all your information will be kept confidential. Here is the drug you are supposed to take. She didn't come with my peer. The work of a peer is to bring us the services.” (TGW, age 22)*

Self‐sampling was viewed favourably compared to facility‐based sampling; however, it was not ideal. Some TGW expressed reservations about the confidentiality of test results because of the involvement of peers—who could be perceived as TGW in the community, thus increasing worry about stigma. If NG/CT self‐tests were available, TGW would prefer to conduct and interpret the tests independently, thus removing peers as intermediaries and maximizing confidentiality.

*“It is not so confidential like the [HIV] self‐testing kit. We collect the sample, but we do not [self]‐test for STIs. There is no way you can [immediately] know whether it is positive or negative. [Peers] bring the sample to the laboratory for interpretation which brings in another hand. The peers do not know how to do [their work] without letting other people know about you. This is in the way they talk, how they behave, and the way they dress. People complain about involuntary disclosure.” (TGW, age 23)*

## 4 DISCUSSION

This qualitative study of TGW enrolled in a cluster randomized trial in Kampala, Uganda, suggests that peer delivery of HIV self‐tests, self‐collection of samples for STI testing and PrEP motivated prevention uptake. Peer support for HIV/STI testing and PrEP adherence was valued by study participants who, in turn, reached out to TGW not engaged in care, taught them about HIVST and encouraged them to take PrEP. HIVST with intimate partners strengthened partnered relationships, but PrEP taking after mutual disclosure of HIV‐negative status aroused suspicions of infidelity. Self‐sampling for STI testing was novel and empowering because it afforded privacy and confidentiality. TGW valued STI testing and treatment as improving their quality of life. Overall, peer services were experienced as extending beyond the provision of HIV prevention commodities, to caring for health and wellbeing by providing psychosocial support and stigma coping strategies as well as facilitating community connectedness.

To our knowledge, this is the first qualitative evaluation of peer‐delivered HIVST, STISS and PrEP for TGW in any setting. As anticipated, TGW peers in our randomized trial acted as recruitment facilitators, health system navigators and intervention deliverers to support prevention uptake by TGW [33]. Peer support improved the uptake of HIV testing by enabling TGW to circumvent the stigma experienced in healthcare settings and from society, thereby mitigating the adverse effects of stigma experiences and practices. This aligns with the Health Stigma and Discrimination Framework, which explains how stigma drivers, facilitators and manifestations impact health‐related outcomes [10, 34]. Our findings agree with studies in the United States which showed that peer navigation improves TGW engagement in HIV care [35, 36]. Implementing peer services in equitable partnership with the studied community may have facilitated community connectedness by empowering TGW, who reached out to hidden members of their community with HIVST and encouraged them to link to PrEP. Our community‐engaged approach included TGW peers in intervention planning and implementation, allowing the inclusion of embodied knowledge in research design and conduct [37]. TGW are disproportionately affected by mental and physical ill health because of intersecting social discrimination and marginalization [37, 38]. We are currently evaluating a multi‐level intersectional stigma reduction intervention to improve PrEP outcomes for TGW in Uganda (R01TW12672). Future efforts to optimize differentiated PrEP delivery for this population should be grounded in collaborative partnerships that build meaningful relationships with transgender communities, acknowledge and share power, and address intersectional causes of health inequities [39].

Self‐sampling for etiological diagnosis of CT and NG was empowering for this sample of TGW. They desired to avoid the embarrassment of exposing their bodies during sample collection at the research clinic. Thus, self‐sampling at home and confidentiality of test results were preferred. Other qualitative research has found that self‐collection of samples is acceptable for TGW in multiple settings [40, 41, 42]. Most TGW in these studies preferred self‐sampling to clinician‐collected samples primarily because of privacy concerns. Our results agree with these findings and suggest that self‐sampling increased utilization of STI testing among TGW for whom avoidance of facility‐based testing is a key barrier to care. A meta‐analysis of 11 studies (*N* = 202,745) from Australia, Denmark and the United States found that self‐collection of samples doubled STI testing uptake and case finding [43]. In the future, self‐STI tests that permit TGW to collect and test their samples and interpret their results privately are needed to maximize testing coverage and linkage to care. Programmes scaling up point‐of‐care HIV/STI testing should be co‐designed with TGW as equal partners to help achieve national and global 95:95:95 HIV targets [12, 28].

HIV self‐tests facilitated mutual partner testing, status disclosure and sexual decision‐making in this sample of TGW. TGW initiated partner testing by asking their partner to self‐test or offering to test them. HIVST is the preferred testing modality of TGW because of its confidentiality and convenience [44]. TGW in other settings have reported that mutual status disclosure strengthened the relationship trust [45, 46]. The dissemination of HIVST to TGW not enrolled in the study extended the reach of HIV testing to a hidden population not otherwise engaged in care due to structural marginalization and the stigmatizing experiences associated with HIV testing in healthcare facilities [44, 47]. Recognizing and addressing the stigma manifestations perpetuating inequitable HIV outcomes will help achieve health justice for underserved TGW [10].

The strengths of our study include the first qualitative evaluation of peer‐delivered HIV self‐tests and STI self‐sampling kits as facilitators of oral PrEP adherence for TGW. We interviewed participants, partners and peers to include multiple perspectives of intervention delivery. The qualitative data enable a detailed understanding of intervention experiences, strengths and drawbacks. One of the authors identifies as transgender, which aided our interpretation of the data. Additionally, we involved transgender colleagues in various aspects, such as reviewing data collection instruments, recruiting and retaining participants, delivering the intervention, reviewing the manuscript and disseminating the results. The limitations of this work include the clinical trial setting, which may not reflect “real world” implementation of HIVST, STISS and PrEP where peer delivery of these interventions is not routine. Like all qualitative research studies, our findings are not, and should not be construed as generalizable. However, they may inform peer delivery of HIV prevention interventions in other African settings.

## 5 CONCLUSIONS

DSD is being scaled up for TGW in sub‐Saharan Africa and elsewhere. Peer support for user‐controlled biomedical interventions may not only benefit individuals but also improve community connectedness and prevention uptake among marginalized populations. Understanding the larger benefits of peer services and integrating them into existing PrEP delivery models could increase prevention coverage and contribute to HIV epidemic control.

## COMPETING INTERESTS

AM reports a grant from Gilead Sciences, Inc outside the submitted work. JEH is a consultant for Merck.

## AUTHORS’ CONTRIBUTIONS

AM and JEH conceptualized the manuscript. JM, VK, BK, GKN, AN and CCT conducted qualitative interviews. MAW, TM and AM supervised qualitative data collection and study implementation. AM, VK, BK, GKN and AN read interview transcripts and coded the data. AM inductively developed categories. AM wrote the first draft along with JEH. MAW, NCW and JEH reviewed drafts and provided substantial edits. RN performed the statistical analyses. All authors read and approved the final version.

## FUNDING

This work was supported through research grants from the United States National Institute of Mental Health (R34MH121084 [AM] and K24MH114732 [JEH]).

## DISCLAIMER

This paper represents the opinions of the authors and does not necessarily represent the official views of the National Institutes of Health.

## Supporting information

Supporting Information

Supporting Information

Supporting Information

## Data Availability

The data that support the findings of this study are available from the corresponding author upon reasonable request.
