# Supplementary material for: “I felt special!”: a qualitative study of peer‐delivered HIV self‐tests, STI self‐sampling kits and PrEP for transgender women in Uganda
Source: J Int AIDS Soc. 2023 Dec 26;26(12):e26201. doi: 10.1002/jia2.26201 (PMC10750840; doi:10.1002/jia2.26201)
Supplement: Supplementary file 1 — Supporting Information [file JIA2-26-e26201-s001.pdf]

Peer Qualitative Interview Guide

Peer Study
Version 1.0
30th September 2021

Introduction: As you know, peers are delivering HIV and STI services to transgender women in the Peer Study. I am talking with you to understand your experiences working as a peer in this study. I am interested in hearing your thoughts and ideas about the peer-delivery program to help us to better understand how and why peer services for trans women may or may not work. This will contribute to developing HIV/STI services that meet the needs of your community. During our discussion today, I will ask you questions that you are free to answer in any way you wish. If a question is unclear to you, please feel free to ask me to explain it.

To begin, I would like to thank you for coming. Tell me a little about yourself.

Tell me a story about how you became a peer?

Now I am going to ask you about your experiences with peer services.

Peer-Delivery: Individual

How would you describe your role in the study?

What did your training to deliver peer services consist of?

Tell me a story of a participant to whom you provided services.

How do you feel about your ability to deliver HIV prevention services to participants?
Probe for details about positive or negative aspects of peer-delivered services.

What challenges do you routinely face as you carry out your duties providing services to your clients?

Do you feel like you can try new things to improve your work as a peer? What new things would you like to do and why?

Can you describe your working relationship with your supervisor at the research clinic? What about your relationships with other peers?

Peer-Delivery: Interpersonal
Tell me more about the experience of interacting with study participants? Are there things you like or do not like?

Tell me a story about delivering HIV self-test kits and PrEP to a client.

How did you find the experience of collecting samples for STI testing?

How do you think peer services are perceived by your participants?

Probe to get as much detail as possible.

Adherence to PrEP can be challenging. What are some of the ways that peers can support adherence for participants?

When your participants come to the study clinic, how long does it usually take for them to receive care? Do they experience any challenges?

What are the most common reasons participants cite for not attending clinic visits?

What are the most common reasons participants cite for not taking PrEP?

Peer-Delivery: Community

How did you feel about participating in research that involves your community?

What do you think are the key challenges faced when delivering HIV/STI services to trans women?

Probe: What barriers exist that limit these services?

What other individuals, groups, or organizations could help to trans women access HIV services?

Overall, how do you and your colleagues feel about the peer-delivery program for trans women? Is it working? Why or why not?

How might these services be changed or organized to better meet the needs and preferences of your community?

How do you think providing peer-delivered HIV prevention services has helped participants to access HIV care?

I do not have any more questions currently. Is there anything else you would like to say to help us understand your experiences of peer services?

Thank you very much for participating in this interview. Do you have any questions about the study or this interview, before we end?

Turn off recorder. Pay close attention to any conversation that occurs after the recorder is turned off.
