# Supplementary material for: “I felt special!”: a qualitative study of peer‐delivered HIV self‐tests, STI self‐sampling kits and PrEP for transgender women in Uganda
Source: J Int AIDS Soc. 2023 Dec 26;26(12):e26201. doi: 10.1002/jia2.26201 (PMC10750840; doi:10.1002/jia2.26201)
Supplement: Supplementary file 2 — Supporting Information [file JIA2-26-e26201-s002.pdf]

Participant Qualitative Interview Guide

Peer Study
Version 1.0
5th July 2020

Introduction
I am speaking to you today to learn about your experiences with testing for HIV, collecting your own samples to test for sexually transmitted infections (STIs) and taking pre-exposure prophylaxis (PrEP). Trans women and their partners are at high risk for HIV and STIs. PrEP is a pill taken every day to prevent someone from getting HIV. Whatever we discuss will remain confidential. Please feel free to share your actual experiences and thoughts. Do you have any questions before we begin?

Background
Let's begin with you telling me something about yourself. For instance, how old are you? What you do to earn a living? Whom do you stay with?

Study Participation
How did it feel when you came to Kasangati for the first time?

What were your expectations? Was the study as you expected? In what ways?

What have you liked about the Peer study?

What have you found challenging about participating in the study?

HIV Self-testing Experiences
Now, I am going to ask you about your experiences with testing for HIV. HIV testing can be done by yourself at home or a place of your convenience. This is called self-testing. The HIV self-test kit uses an oral swab to test for the body's response to fighting HIV. It is painless and easy to use. No blood is required.

When you began the Peer study, you were given HIV self-test kits to use at home. Tell me a story about how you test yourself for HIV.

Probe for details about experiences and perceptions of HIV self-testing

What was using the self-test kit like for you? What was easy? What was challenging?

Probes: How did you find the instructions on how to use the kit?

How did you interpret the result?

What was your reaction(s) to the HIV self-test result?

Probe: How did you feel when you found out your HIV status?

How does using a self-testing kit compare to HIV testing at the clinic?

Probe for advantages and disadvantages of self-testing.

Please describe what you do to protect yourself from HIV?

Probe in detail about condom use and/or PrEP use

<u>Next, I am going to ask you about HIV self-testing for your sexual partner(s)</u>

If you have a regular partner(s), tell me the story of how you introduced the self-testing kit to your partner.

Probe: How did you feel when you talked to your partner about testing?

After you gave the kit to your partner(s), what happened? How did they react when given the kit?

Probe in depth

Did you test your partner, or did they test themselves? Explain how the testing was done.

How did you get to know your partner's HIV status? What happened after knowing partner's status?

In what ways has self-testing your partner(s) influenced your decisions on whom to have sex with?

Probe to get as much detail as possible. How did self-testing change decision making around using condoms?

STI Testing Experiences

Now I am going to ask you about testing for STIs. Transwomen say that sexually transmitted infections (STIs) are common in their community, but they fear going to clinics for testing. However, it is now possible to collect your own samples to test for STIs.

What do trans women in your community say about sexually transmitted infections?

How do you feel about testing for sexually transmitted infections?

Probe for details about STI testing experiences

What are your concerns

Probe about the experience of collecting samples for STI testing, confidentiality, receipt of results

What do you think about using peers to bring test kits to your home? What have you liked or disliked about this strategy of using peers?

Probe for details about concerns regarding confidentiality, stigma, fear of disclosure

What are likely to be the best ways to improve self-collection of STI samples?

Probe for details about perceptions of collecting own samples for STI testing and whether sexual partners would also be interested collecting their STI samples

What helped you test yourself or collect STI samples?

Encourage participants to enumerate successes or challenges with using video, pictures, or peer support to understand how to test themselves

PrEP Perspectives

Now I am going to ask you about taking PrEP pills.

Tell me a story about a time when you felt that PrEP did or did not work for you.

How well do you think you have been taking the pills?

Probes: Ask about reminders and activities of daily living that could remind to take PrEP.

What challenges do you face in taking the pills? Tell me about some of them.

Probe to get as much detail as possible.

What would you tell/advise someone finding it difficult to taking their pills?

Probe to get as much detail as possible.

Is there anything else you would like to say to help us understand your experience of HIV and STI testing and/or taking PrEP pills?

Peer Delivery

This study uses peers to bring test kits and PrEP to your home or a place of your convenience.

How did you feel the first time the peer visited you?

What is good about this method?

What problems do you have with this method? Why?

Tell me a story about when you felt that peer visits did or did not work for you.

Is there anything else you would like me to know about using peers to deliver testing kits and PrEP to you?

Closing

I do not have any more questions at this time. Thank you very much for participating in this interview. Before we end, is there anything else you would like to tell me?

Thank you for your time.

Turn off recorder. Pay close attention to any conversation that occurs after the recorder is turned off.
